# Single-cell transcriptomics combined with Mendelian randomization reveals the pivotal role of *WDR83OS* in clear cell renal cell carcinoma: An observational and bioinformatic study

**DOI:** 10.1097/MD.0000000000050539

**Published:** 2026-09-18

**Authors:** Leting Xiao, Aimureguli ·Yusan, Yongde Cao, Fangqian Yue, Jiaxin Feng, Huayan Wu, Lingling Sun, Junbo Qiu, Yinhao Xiao, Shilin Zhang

**Affiliations:** a Department of Surgery, Foshan Maternal and Child Healthcare Hospital Affiliated to Guangdong Medical University, Foshan, Guangdong Province, China; b School of Information Science, Guangdong University of Finance and Economics, Guangzhou, Guangdong Province, China.

**Keywords:** clear cell renal cell carcinoma, Mendelian randomization, single-cell RNA-sequencing, *WDR83OS*

## Abstract

This study aimed to comprehensively investigate the molecular mechanisms of clear cell renal cell carcinoma (ccRCC), identify key cellular subpopulations and genes, develop an effective diagnostic model, and screen potential targeted therapies for ccRCC. We analyzed single-cell transcriptomic sequencing data to identify the major cellular subpopulations in ccRCC. High-dimensional weighted gene co-expression network analysis and multiple machine learning algorithms were used to identify key genes and develop a diagnostic model. Two-sample Mendelian randomization analysis was performed to assess causality. Molecular docking was used to identify a candidate therapeutic agent. Data processing was conducted using R and Python. The proportion of endothelial cells was significantly higher in ccRCC (*P* < .001). High-dimensional weighted gene co-expression network analysis showed that the pink module was closely associated with endothelial cells. Univariate logistic regression and Least Absolute Shrinkage and Selection Operator identified 11 key genes: *WDR83OS, TMA7, PFDN5, DSTN, PHPT1, HMGN3, TMSB10, RPL27A, RPL23A, RPL15,* and *RPL27*. Based on these genes, a diagnostic model for ccRCC was developed using multiple machine learning algorithms and achieved an area under the receiver operating characteristic curve of 0.960. In addition, 2-sample Mendelian randomization analysis supported a causal association between *WDR83OS* and ccRCC (inverse-variance weighted: odds ratio = 1.160, *P* = .033). Molecular docking indicated that oxyphenbutazone had a high binding affinity for *WDR83OS*, with a binding energy of −7.124 kcal/mol. By integrating multiple bioinformatic approaches, this study identified key cellular populations and genes in ccRCC, developed a reliable diagnostic model, and highlighted WDR83OS as a potentially important therapeutic target.

## 1. Introduction

Kidney cancer is an increasingly important global health concern. Its incidence has continued to rise over the past several decades, and it is now among the 10 most common cancers, particularly in Western countries.^[1]^ Renal cell carcinoma (RCC) is the most common histological type, of which ccRCC accounts for 70% to 80%.^[1,2]^ Advanced ccRCC has a poor prognosis, with a 5-year survival rate below 10%, owing to frequent recurrence, a high propensity for metastasis, and heterogeneous treatment responses.^[3,4]^ A more detailed understanding of tumor molecular biology and the complex tumor microenvironment (TME) is therefore needed to improve treatment and advance individualized care.

ccRCC is characterized by substantial tumor heterogeneity and is strongly influenced by genetic alterations.^[5,6]^ Its pathogenesis involves genetic changes that disrupt cellular metabolism, including the Warburg effect, and Von Hippel-Lindau mutations are a hallmark of the disease.^[5,7]^ Additional mutations in Polybromo 1, BRCA1-associated protein 1, and SET Domain Containing 2 also contribute to disease progression.^[8–10]^ Non-clear cell RCC (nccRCC) accounts for 20% to 30% of RCCs and includes papillary RCC (characterized by MET proto-oncogene, receptor tyrosine kinase activation), chromophobe RCC (characterized by mitochondrial abnormalities and relatively slow growth), translocation RCC, and rarer subtypes such as collecting duct carcinoma.^[2,5,11]^ These subtypes generally have poorer outcomes and fewer treatment options. Sarcomatoid differentiation occurs in approximately 5% of RCCs and is associated with highly aggressive behavior.^[3,11]^ Distinguishing benign from malignant renal neoplasms can also be challenging; for example, molecular testing for BRAF alterations may assist in diagnosing metanephric adenoma.^[12]^

Treatment of metastatic RCC has shifted from cytokine-based therapies such as interferon alpha to vascular endothelial growth factor–targeted agents (e.g., sunitinib), mechanistic target of rapamycin inhibitors (e.g., everolimus), and immune checkpoint inhibitors (e.g., nivolumab).^[13]^ The International Metastatic RCC Database Consortium model is used for risk stratification and treatment planning.^[6,13]^ Programmed cell death protein 1/programmed death-ligand 1 inhibitors are standard treatments for ccRCC; however, evidence in nccRCC remains limited and heterogeneous. Agents such as cabozantinib have shown activity in selected subtypes, including papillary RCC, and combinations with immunotherapy have demonstrated promising results.^[14–16]^ Further clinical trials are needed to evaluate surgery and additional combination therapies in nccRCC.^[11,13]^

Previous studies have predominantly used bulk tissue samples, which combine signals from multiple cell populations and therefore obscure intratumoral heterogeneity, particularly the genetically distinct ccRCC subclones.^[6]^ In recent years, single-cell RNA sequencing (scRNA-seq) has emerged as a valuable tool for characterizing cellular composition and investigating interactions between tumor cells and the surrounding microenvironment.^[17–19]^ Mendelian randomization (MR) uses genetic variants as instrumental variables to infer causal relationships and provides a complementary approach to investigating disease etiology.^[20,21]^

This study integrates scRNA-seq, weighted gene co-expression network analysis, bulk RNA-sequencing, and MR to characterize endothelial cell (EC) heterogeneity in ccRCC and its role in disease progression, identify core genes, and develop a diagnostic model. Extension of this framework to nccRCC may provide further insights into early detection, individualized treatment, and improved kidney cancer care.

## 2. Materials and methods

### 2.1. Single-cell transcriptomic data analysis

We analyzed the publicly available scRNA-seq datasets GSE159115 and GSE237425, obtained from the Gene Expression Omnibus (https://www.ncbi.nlm.nih.gov/geo/). The Seurat package (version 5.2.1; Satija Lab, New York Genome Center) was used for quality control, data standardization and normalization, identification of highly variable genes, dimensionality reduction, clustering, and cell-type annotation. Batch effects between datasets were corrected using Harmony (version 1.2.3; Immunogenomics, Broad Institute of MIT and Harvard) to improve cross-dataset comparability and the reliability of downstream analyses.

### 2.2. High-dimensional weighted gene co-expression network analysis

To characterize gene-expression patterns and regulatory networks at single-cell resolution, high-dimensional weighted gene co-expression network analysis (hdWGCNA) was used to standardize and reduce the dimensionality of the single-cell gene-expression data. This approach reduced technical variation while preserving biological variation. A gene co-expression network was constructed using soft-thresholding, and approximate scale-free topology was assessed to support the biological and statistical validity of the network. Functionally related gene clusters were identified using a topological overlap measure to characterize potential gene regulatory network patterns.

### 2.3. Acquisition of bulk transcriptomic data

Bulk RNA-sequencing data for ccRCC were obtained from The Cancer Genome Atlas (TCGA). Data were retrieved on February 27, 2025, using the search terms “TCGA-KIRC” and “transcriptome profiling.” A total of 614 samples were included: 541 tumor samples and 73 normal samples.

### 2.4. Screening of core genes and development of diagnostic models

Core genes in the pink module were first screened using univariable logistic regression and Least Absolute Shrinkage and Selection Operator. Several machine learning algorithms were then used to build diagnostic models based on the selected key genes, including k-nearest neighbors, logistic regression, support vector machine, linear discriminant analysis, naive Bayes, random forest, and recursive partitioning and regression trees. For model validation, an independent external dataset was obtained from the TCGA repository. The acquired data were divided into training and validation sets at a 70:30 ratio to support methodological rigor and reproducibility. Ten repeats of 5-fold cross-validation were performed on the training set to identify the best-performing model. The selected model was subsequently evaluated in the validation set to assess its performance and generalizability.

### 2.5. MR analysis between *WDR83OS* and ccRCC

We performed an MR analysis to investigate the relationship between *WDR83OS* and ccRCC. Genome-Wide Association Study (GWAS) summary statistics for *WDR83OS* were retrieved from the IEU OpenGWAS platform (http://gwas.mrcieu.ac.uk/), and genetic data for ccRCC were obtained from the FinnGen consortium. All statistical analyses were conducted using the TwoSampleMR package in R. Valid instrumental variables were required to satisfy 3 assumptions: relevance, whereby the genetic instrument is associated with the exposure; independence, whereby the instrument is unrelated to confounding variables; and exclusion restriction, whereby the instrument affects the outcome only through the exposure. Single nucleotide polymorphisms (SNPs) strongly associated with the exposure were selected as instruments at *P* < 5 × 10^−8^. Linkage disequilibrium was addressed using thresholds of *R*^2^ < 0.1 and a clumping distance of 10,000 kb. *F*-statistics >10 were required to minimize weak-instrument bias. The 2-sample MR analysis included 5 complementary methods, with inverse-variance weighted (IVW) designated as the primary approach. Secondary analyses included MR-Egger regression, weighted-median estimation, simple-mode estimation, and weighted-mode estimation.

### 2.6. Molecular docking

Molecular docking was performed to identify drugs that could potentially target and inhibit *WDR83OS* in ccRCC. Structural information for the active compounds was obtained from the Compound Library and imported into ChemBio3D 14.0 (PerkinElmer Informatics, Inc.), where the spatial configurations were energy-minimized before export in mol2 format. Following processing in AutoDockTools 1.5.6 (Molecular Graphics Laboratory, The Scripps Research Institute), the 3-dimensional crystal structure of the target protein was obtained from UniProt (UniProt Consortium). Water molecules and organic residues were removed from the target protein structure using Notepad2 (Florian Balmer, Flo’s Freeware), after which the structure was loaded into AutoDockTools 1.5.6 for hydrogen addition, charge assignment, and atom-type assignment. Molecular docking simulations were conducted using AutoDock Vina (The Scripps Research Institute), and the results were visualized and analyzed using PyMOL 2.6.1 (Schrödinger, LLC).^[22–24]^

### 2.7. Ethics statement

This study used only publicly available, de-identified, summary-level data from the Gene Expression Omnibus, TCGA, and FinnGen databases. Because no participant-level interventions or identifiable personal data were involved, institutional review board approval and informed consent were not required. Ethical oversight and participant consent were obtained and reported by the investigators of the original studies.

## 3. Results

### 3.1. Single-cell transcriptomic analysis and EC subtyping

**Figure 1.**
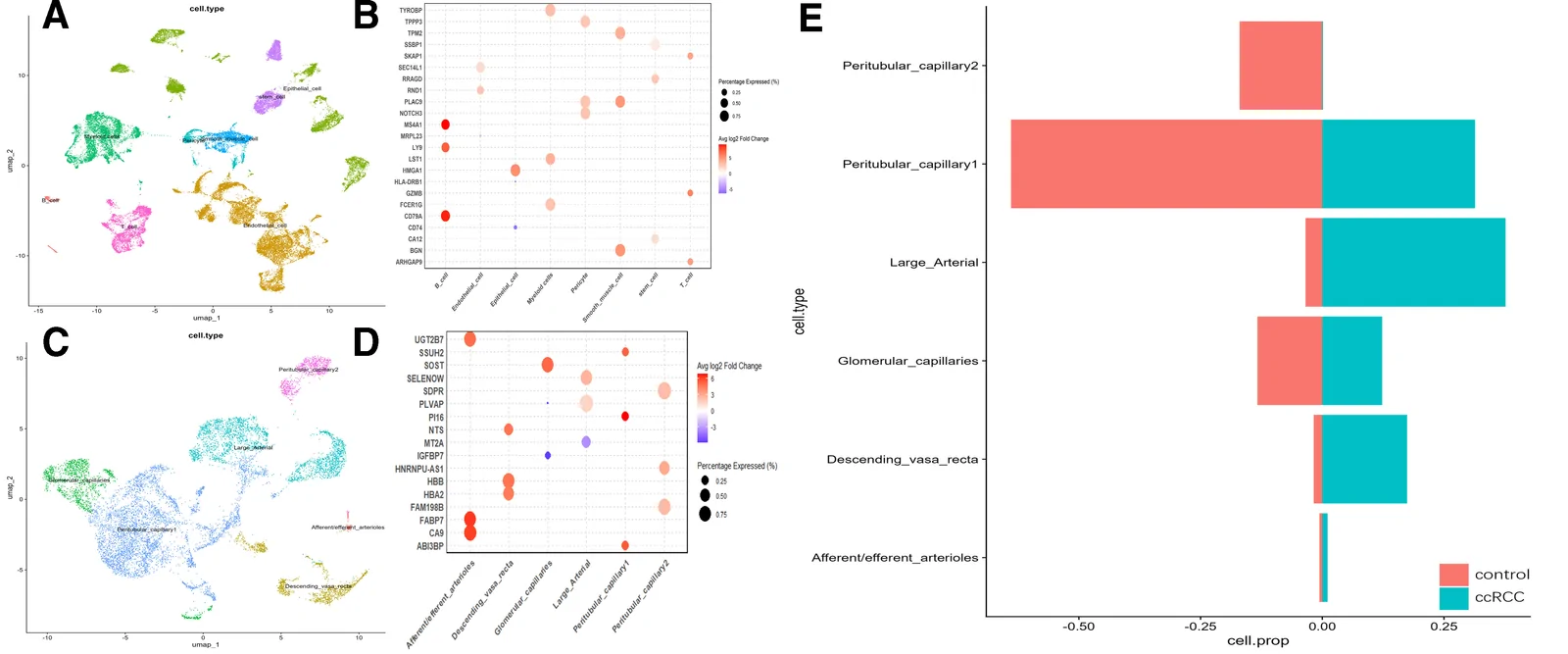
Single-cell transcriptomic sequencing data analysis reveals changes in the proportion of endothelial cells in ccRCC. (A) UMAP plot of primary clustering cell types; (B) bubble plot showing the expression of marker genes for different cell types in primary clustering; (C) UMAP plot of different endothelial cell subtypes; (D) heatmap displaying the expression of marker genes across different endothelial cell subtypes; (E) bar plot illustrating the proportional changes in the composition of different endothelial cell subtypes between groups. ccRCC = clear cell renal cell carcinoma, UMAP = Uniform Manifold Approximation and Projection.

GSE159115 included 8 tumor samples and 6 normal samples, whereas GSE237425 included 4 tumor samples and 4 normal samples. After quality control, approximately 32,887 cells were retained for downstream analysis. Batch effects were corrected, followed by dimensionality reduction, cell clustering, and cell-type annotation. Uniform Manifold Approximation and Projection identified the major cell populations, including ECs, T cells, epithelial cells, B cells, myeloid cells, stem cells, pericytes, and smooth muscle cells (Fig. 1A). Marker expression for these populations is shown in Figure 1B. Given the critical roles of ECs in ccRCC initiation and progression,^[25]^ ECs were isolated for further subpopulation and disease-associated analyses.^[26]^ Six EC subtypes were identified: Peritubular_capillary1, Peritubular_capillary2, Glomerular_capillaries, Descending_vasa_recta, Large_Arterial, and Afferent/Efferent_arterioles (Fig. 1C). Marker-gene expression patterns across these subtypes are shown in Figure 1D. Comparison of cell proportions between groups revealed a marked increase in Large_Arterial ECs in tumor samples (Fig. 1E).

### 3.2. Identification and functional characterization of the pink module

**Figure 2.**
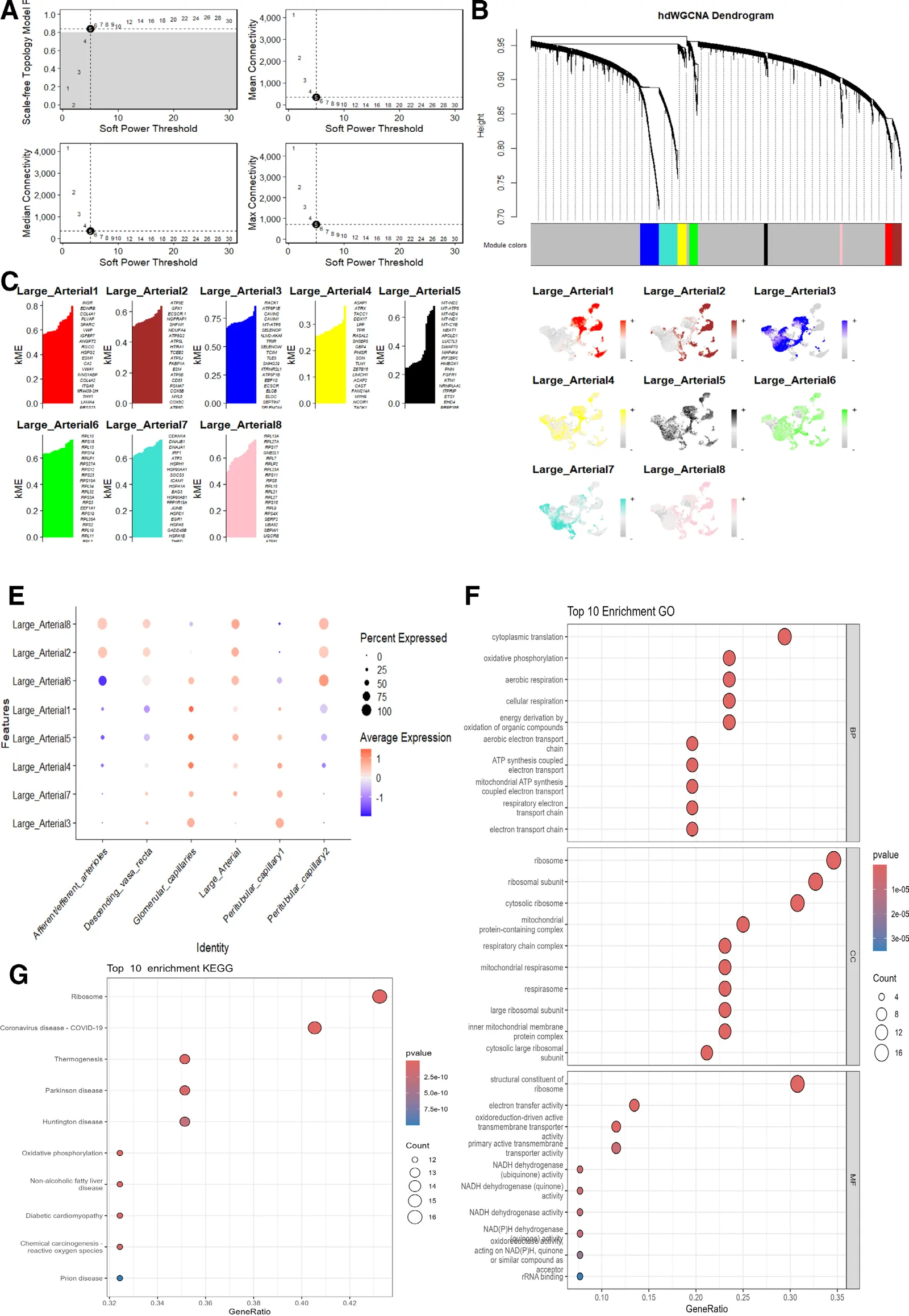
Identification of key gene modules in endothelial cells. (A) Soft threshold value; (B) hierarchical clustering dendrogram of the hdWGCNA; (C) top 20 genes with the highest correlation within different modules; (D) UMAP plot of gene scores across different modules; (E) dot plot of gene scores for different modules; (F, G) results of GO and KEGG enrichment analysis for genes in the pink module. HdWGCNA = high-dimensional weighted gene co-expression network analysis, GO = Gene Ontology, KEGG = Kyoto Encyclopedia of Genes and Genomes, UMAP = Uniform Manifold Approximation and Projection.

Our preceding analysis indicated that Large_Arterial ECs were strongly associated with ccRCC. To identify major gene modules in these cells from ccRCC samples, Large_Arterial cells were isolated and analyzed using hdWGCNA. Cells were aggregated into metacells, and a soft-thresholding power of 5 was used to identify 8 gene modules (Fig. 2A and B). The top 20 hub genes in each module are shown in Figure 2C. Module scores were calculated with Seurat’s AddModuleScore function using the genes within each module and visualized using Uniform Manifold Approximation and Projection (Fig. 2D) and a dot plot (Fig. 2E). The Large_Arterial8 group, which was closely associated with disease, showed the highest expression of pink-module genes (Fig. 2E), suggesting that these genes may be important in Large_Arterial ECs in ccRCC. Genes in the pink module were subsequently subjected to Gene Ontology and Kyoto Encyclopedia of Genes and Genomes enrichment analyses using the clusterProfiler (YuLab, Southern Medical University) package in R (Fig. 2F and G). The enrichment results indicated that these genes may contribute to ccRCC through pathways related to ribosomal protein synthesis, mitochondrial energy metabolism and ATP production, thermogenesis, and reactive oxygen species–mediated cellular damage. Kyoto Encyclopedia of Genes and Genomes analysis additionally linked these genes to pathways involved in viral infections, including COVID-19; neurodegenerative disorders, including Parkinson and Huntington diseases; metabolic and cardiovascular disorders, including fatty liver disease and diabetes-related heart disease; oxidative processes; chemical carcinogenesis; and energy metabolism.

### 3.3. Identification of core genes and construction of diagnostic models using multiple machine learning algorithms

**Figure 3.**
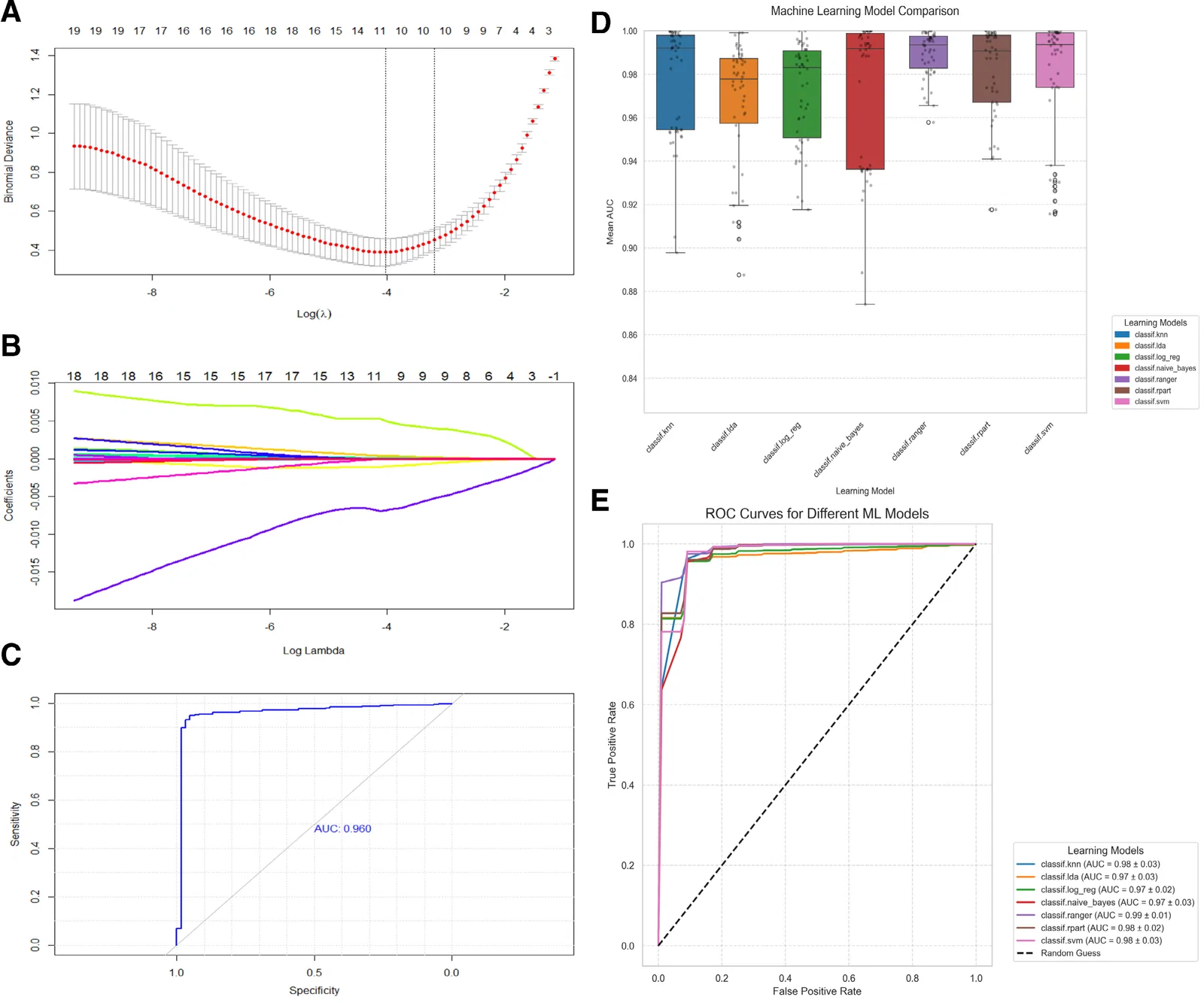
Acquisition of key genes based on ML and construction of diagnostic models. (A) Coefficient profile for gene number reduction; (B) penalty plot of the LASSO model; (C) diagnostic performance of the rRanger model on an external validation set; (D, E) diagnostic performance of 7 different models. AUC = area under the receiver operating characteristic curve, LASSO = Least Absolute Shrinkage and Selection Operator, ML = machine learning, ROC = receiver operating characteristic.

To identify core genes within the pink module, univariable logistic regression and Least Absolute Shrinkage and Selection Operator analyses were performed using genes from the TCGA-KIRC dataset (Fig. 3A and B). These analyses identified 11 genes: *WDR83OS, TMA7, PFDN5, DSTN, PHPT1, HMGN3, TMSB10, RPL27A, RPL23A, RPL15,* and *RPL27*. Based on these genes, diagnostic models were constructed using k-nearest neighbors, logistic regression, support vector machine, linear discriminant analysis, naive Bayes, random forest, and recursive partitioning and regression trees. The TCGA-KIRC dataset was divided into training and validation sets at a 7:3 ratio. Because normal tissue samples were limited in TCGA, we evaluated the sample distribution and performed balanced subsampling of the training set to reduce overfitting. Model robustness was assessed using 10 repeats of 5-fold cross-validation. All models showed robust predictive performance (Fig. 3D and E). The rRanger algorithm provided the best discrimination between normal tissue and ccRCC (Fig. 3D) and was therefore selected as the final model. Evaluation in the validation set yielded an area under the receiver operating characteristic curve of 0.960, indicating high diagnostic performance (Fig. 3C).

### 3.4. Two-sample MR analysis of *WDR83OS* and ccRCC

**Figure 4.**
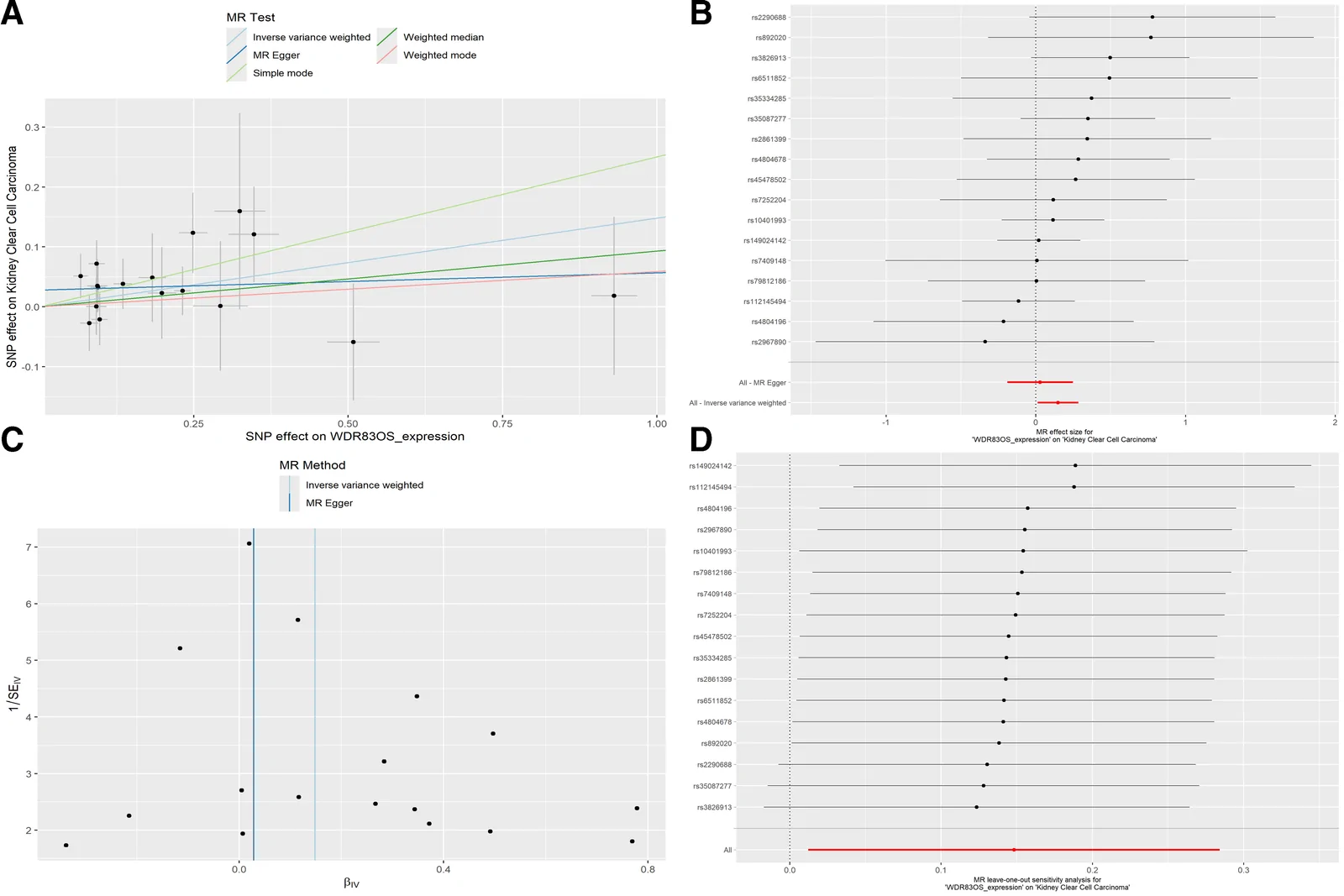
Results of 2-sample MR analysis of *WDR83OS* and ccRCC. (A) Scatter plot showing the causal effect of *WDR83OS* on ccRCC; (B) forest plot illustrating the causal effects of individual SNPs on ccRCC risk; (C) funnel plot visualizing the overall heterogeneity of MR estimates of *WDR83OS* on ccRCC; (D) leave-one-out sensitivity analysis visualizing the causal effect of *WDR83OS* on ccRCC risk when excluding one SNP at a time. ccRCC = clear cell renal cell carcinoma, MR = Mendelian randomization, SNP = single nucleotide polymorphism.

*WDR83OS* was a prominent gene in the pink module. To investigate a potential causal relationship between *WDR83OS* and ccRCC, we conducted 2-sample MR analysis. GWAS data for *WDR83OS* were retrieved, focusing on cis-acting SNPs from eqtl-a-ENSG00000105583. Linkage disequilibrium pruning at a distance of 10,000 kb and *R*^2^ = 0.1 yielded 17 SNPs, which were evaluated using multiple MR methods. The IVW analysis indicated a positive association (odds ratio = 1.160, *P* = .033; Table 1; Fig. 4A and B). Cochran’s *Q* test did not indicate significant heterogeneity (*P* = .843 for MR-Egger and *P* = .779 for IVW). The MR-Egger intercept test did not indicate evidence of horizontal pleiotropy (*P* = .192). The funnel plot showed a symmetrical distribution of *WDR83OS* variants with limited heterogeneity (Fig. 4C). Leave-one-out analysis indicated that no individual SNP drove the association, and the IVW confidence interval remained above zero. Collectively, the IVW results suggest that higher *WDR83OS* expression is associated with an increased risk of ccRCC (Fig. 4D).

**Table 1:** Detailed estimation results of 5 MR methods.

Method	nsnp	*b*	se	*p*val	or	or_lci95	or_uci95
Simple mode	17	0.250	0.171	0.162	1.285	0.919	1.796
Inverse-variance weighted	17	0.148	0.069	0.033	1.160	1.012	1.329
MR-Egger	17	0.029	0.112	0.800	1.029	0.827	1.281
Weighted median	17	0.093	0.099	0.350	1.097	0.903	1.334
Weighted mode	17	0.059	0.112	0.608	1.061	0.851	1.322

MR = Mendelian randomization.

### 3.5. Oxyphenbutazone as a potential inhibitor of WDR83OS in ccRCC

**Figure 5.**
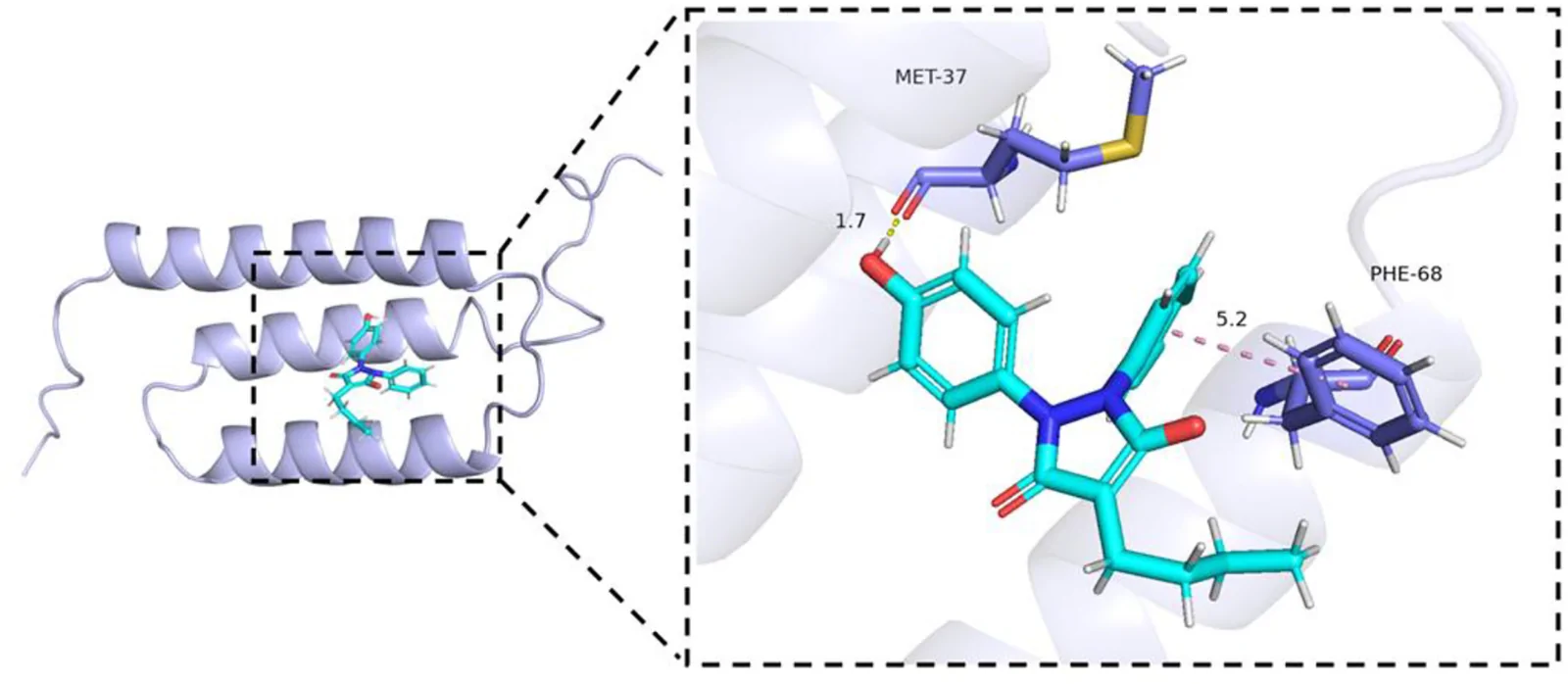
Molecular docking results of oxyphenbutazone and *WDR83OS*. The blue structure represents the *WDR83OS* protein, and the cyan molecule is oxyphenbutazone. Key amino acid residues are displayed in stick representation, hydrogen bonds are indicated by yellow dashed lines, and Pi-Pi interactions are shown in pink.

The preceding analyses indicated that *WDR83OS* may play an important role in ccRCC. To identify potential inhibitors, the Food and Drug Administration Western Drug Compound Library was screened, and oxyphenbutazone was identified as a candidate inhibitor of *WDR83OS* (Methods). AutoDock Vina was used to simulate the interaction between oxyphenbutazone and the *WDR83OS* protein. The calculated binding energy was −7.124 kcal/mol. Oxyphenbutazone formed one hydrogen bond with MET-37 and one hydrogen bond with PHE-68. These interactions may contribute to the stability of the protein–ligand complex and thereby influence biological activity (Fig. 5).

## 4. Discussion

This study provides a detailed characterization of gene expression in ECs within the ccRCC TME, offering insights into ccRCC progression and potential therapeutic strategies. ECs were particularly abundant in the Large_Arterial subgroup and exhibited metabolic alterations. These findings are consistent with growing evidence that the TME promotes ccRCC progression. hdWGCNA identified a pink module enriched in genes related to oxidative phosphorylation and ribosome biogenesis, suggesting that ECs may actively remodel the TME through increased mitochondrial energy production and protein synthesis. Other single-cell studies of kidney and ccRCC vasculature have reported similar metabolic reprogramming,^[26,27]^ which may support rapid tumor growth while promoting an immunosuppressive microenvironment. Collectively, endothelial-cell metabolic activity may contribute to ccRCC progression.

Building on these findings and addressing the clinical need for improved diagnostic approaches,^[28]^ we developed a machine learning model based on 11 genes. The model achieved an area under the receiver operating characteristic curve of 0.960 and outperformed conventional markers. The identification of tumor-specific markers through scRNA-seq is an important direction in contemporary cancer research.^[17,18]^ The inclusion of several ribosomal protein genes, including *RPL27A* and *RPL23A*, in our signature is consistent with the findings of Guimaraes et al^[29]^ and supports the hypothesis that ccRCC depends on enhanced regulation of protein translation.

Among these biomarkers, *WDR83OS* emerged as a particularly compelling candidate driver of ccRCC. To our knowledge, *WDR83OS* has not previously been linked to cancer; its reported clinical associations are limited to non-neoplastic conditions, including pediatric cholestatic liver disease^[30]^ and a neurodevelopmental disorder.^[31]^ These conditions are distinct from ccRCC. Using MR, an increasingly applied approach for causal inference in cancer research,^[20,21]^ we provide initial evidence that higher *WDR83OS* expression is associated with an increased risk of ccRCC (IVW: odds ratio = 1.16, *P* = .033). *WDR83OS* encodes Asterix, a membrane-associated molecular chaperone, and may promote tumorigenesis through several mechanisms: facilitating the folding and activation of membrane proteins involved in pathways such as phosphoinositide 3-kinase/protein kinase B; interacting with ribosomes to increase protein synthesis required for growth; contributing to endoplasmic reticulum stress and DNA damage when absent; and cooperating with tumor-promoting factors such as *TMSB10* to enhance cell migration and survival.^[32]^

To translate these molecular findings into a potential therapeutic strategy, we identified oxyphenbutazone as a candidate inhibitor of *WDR83OS*. A virtual high-throughput screen of approximately 1500 Food and Drug Administration–approved compounds was performed to identify drugs that could potentially be repurposed for ccRCC. Compounds were docked against a modeled and optimized 3-dimensional structure of *WDR83OS* using AutoDock Vina^[22–24]^ and ranked by predicted binding affinity. Using a binding-energy cutoff of −6.5 kcal/mol, oxyphenbutazone showed the most favorable predicted binding affinity, with a binding energy of −7.124 kcal/mol, and was therefore selected for further consideration. Oxyphenbutazone is also an nonsteroidal anti-inflammatory drug that targets cyclooxygenase-2 and modulates inflammation.^[33,34]^ Given the roles of inflammation and oxidative damage in the ccRCC TME, we hypothesize that oxyphenbutazone may exert antitumor effects by targeting *WDR83OS* and favorably altering the cellular microenvironment.

Our integrated multi-omics and computational approach provides important insights; however, the findings remain based on statistical and in silico evidence. Experimental validation is therefore required before clinical translation. Future in vitro studies should use approaches such as CRISPR-Cas9-mediated *WDR83OS* knockdown in ccRCC cells to assess the effects of oxyphenbutazone on cell growth and apoptosis. Subsequent in vivo studies in mouse tumor-graft models will be needed to evaluate its therapeutic potential.

In conclusion, this study demonstrates that metabolic alterations in ECs may support ccRCC progression, identifies *WDR83OS* as a novel candidate contributor to ccRCC, and proposes the readily available drug oxyphenbutazone as a potential therapeutic agent. The study provides a robust diagnostic framework and a foundation for developing therapies targeting the *WDR83OS* pathway in ccRCC.

## Author contributions

**Data curation:** Aimureguli ·Yusan, Fangqian Yue, Jiaxin Feng, Huayan Wu, Junbo Qiu.

**Visualization:** Lingling Sun.

**Methodology:** Yinhao Xiao, Shilin Zhang.

**Writing – original draft:** Leting Xiao, Aimureguli ·Yusan, Yongde Cao.
